# Laceration of Perineum and Rectum

**Published:** 1871-09

**Authors:** Irael B. Washburn

**Affiliations:** Star City, Indiana


ARTICLE III.—*Laceration of Perineum and Rectum.* By IRAEL B. WASHBURN, M.D., Star City, Indiana.

I was called to see W. H. B., August 24th, 1870. Upon inquiry I learned that in sliding off a hay stack that he had just completed, he struck upon the end of a fork handle. The tines of the pitchfork were set in the ground, and the top of the handle was resting against the stack. The inner part of the right ischiatic protuberance struck upon the end of the handle, his body fell to the right, the handle perforating the skin, and, upon examination, I found it passed through the perineal fascia and lacerating the rectum half an inch or more above the internal sphincter.

I cleansed the wound as well as possible, applied warm water dressings, and gave an opiate astringent to control the action of the bowels. The 26th I found my patient free from pain, and, to use his own expression, very comfortable. On the 28th I ordered an enema of warm soap suds, which produced a free evacuation, the fecal matter passing through the wound. At this time some of the patient's officious friends were clamorous for "another doctor." I consulted Dr. Fitch, of Logansport, who advised me to divide the sphincters and all tissues below the injury. I also consulted Prof. Powell, of Rush Medical College, who advised that the operation be performed. I submitted the advice of Doctors Fitch and Powell to my patient, who is an intelligent young farmer. He chose to "wait and see what nature would do." The wound healed to the size of a goose quill in about eight weeks, and remained in that condition until the February following, when it completely closed up, and is now quite well.

After the first six weeks Mr. B. went to work, and suffered no particular inconvenience from the fistula that remained unhealed so long.

